# A Systematic Review of using Virtual and Augmented Reality for the Diagnosis and Treatment of Psychotic Disorders

**DOI:** 10.1007/s40501-023-00287-5

**Published:** 2023-06-14

**Authors:** Lucy Lan, Jennifer Sikov, Julia Lejeune, Chelsea Ji, Hannah Brown, Kim Bullock, Andrea E. Spencer

**Affiliations:** 1 Department of Psychiatry and Behavioral Sciences, Stanford University School of Medicine, 401 Quarry Rd, Stanford, CA 94304 USA; 2 Department of Psychology, Florida International University, Miami, Fl USA; 3 Department of Psychology, University of Illinois Chicago, Chicago, IL USA; 4 Chronic Traumatic Encephalopathy Center, Boston University, Boston, MA USA; 5 Department of Psychiatry, Boston Medical Center, Boston, MA USA; 6 Department of Psychiatry and Behavioral Health, Northwestern University Feinberg School of Medicine, Chicago, IL USA

**Keywords:** Virtual reality, Metaverse, Augmented reality, Psychosis, Treatment, Digital therapeutics

## Abstract

**Objective:**

Immersive virtual reality (VR) and augmented reality (AR) have the potential to improve the treatment and diagnosis of individuals experiencing psychosis. Although commonly used in creative industries, emerging evidence reveals that VR is a valuable tool to potentially improve clinical outcomes, including medication adherence, motivation, and rehabilitation. However, the efficacy and future directions of this novel intervention require further study. The aim of this review is to search for evidence of efficacy in enhancing existing psychosis treatment and diagnosis with AR/VR.

**Methods:**

2069 studies involving AR/VR as a diagnostic and treatment option were reviewed via PRISMA guidelines in five databases: PubMed, PsychInfo, Embase, and CINAHL.

**Results:**

Of the initial 2069 articles, 23 original articles were eligible for inclusion. One study applied VR to the diagnosis of schizophrenia. Most studies demonstrated that the addition of VR therapies and rehabilitation methods to treatment-as-usual (medication, psychotherapy, social skills training) was more effective than traditional methods alone in treating psychosis disorders. Studies also support the feasibility, safety, and acceptability of VR to patients. No articles using AR as a diagnostic or treatment option were found.

**Conclusions:**

VR is efficacious in diagnosing and treating individuals experiencing psychosis and is a valuable augmentation of evidence-based treatments.

**Supplementary Information:**

The online version contains supplementary material available at 10.1007/s40501-023-00287-5.

## Introduction

Virtual Reality (VR) and Augmented Reality (AR) are emerging technologies that have the potential to enhance existing means of diagnosing and treating mental health disorders [1–6]. VR is an immersion experience that shuts out the physical world (e.g. using a 360° head-mounted display [7]. AR overlays virtual objects on the real-world environment (e.g. popular social media platforms’ features such as Snapchat lenses and Pokémon Go) [8]. Using AR/VR, a patient’s reality can be explored, expanded, and challenged. Visual, auditory, haptic, somatosensory, and olfactory stimuli can be applied to enhance general wellness, encourage learning, provide entertainment value, and target aberrant behaviors or cognitive patterns [2, 5].

The concept of VR was first formulated in the 1960’s and the first commercial tools appeared on the market in the 1980’s [9]. While current applications are mostly in the creative industries of gaming, entertainment, and retail, VR has also expanded into the healthcare industry, with uses ranging from medical imaging and surgical collaboration to medical education [9–14]. VR has been used as a tool for patient care across a broad array of specialties, including stroke rehabilitation [15], balance support in adults with Parkinson’s disease [16], upper extremity functioning training in children with cerebral palsy [17], and pain management during childhood immunizations [18]. AR emerged in the 1990's as an enhancement to the completely synthetic environment of VR, incorporating video feeds from the real world to augment the animated VR [19, 20]. Medical applications of AR have largely concentrated on improving surgical visualization providing live, 3D datasets from noninvasive imaging (MRI/CT/ultrasound), superimposed on the patient laying on the operative table [20]. AR and VR are collectively referred to as the “metaverse” and are examples of digital therapeutics, evidence-based treatments driven by software programs.

In the past two decades, a growing body of literature has emerged which explores AR/VR applications in psychiatry. For anxiety disorders, VR has been shown to be a useful tool in therapist-supported exposure therapy, particularly with patients experiencing symptoms of specific phobia [21], social anxiety [22], or PTSD [23]. AR/VR has also been applied to treat autism spectrum disorder (ASD) [24], eating disorders [25], attention deficit/hyperactivity disorder (ADHD) [26], and substance-use disorders [3]. For example, in a study with 30 children with high-functioning ASD, social skills training was provided via a VR platform over 5 weeks with significant improvements in emotion recognition, attention, and executive function [24].

Treating and diagnosing schizophrenia and related psychosis-spectrum conditions could be one very important application of AR/VR technology in mental health. Schizophrenia is a severe mental illness characterized by a loss of connection with reality, which can cause profound suffering for patients and their families [27]. Diagnosis of schizophrenia relies on a largely subjective clinical diagnostic assessment. Although antipsychotic medications can often effectively treat positive psychotic symptoms (e.g. hallucinations, delusions), these medications do not treat the negative symptoms (e.g. social withdrawal, anhedonia, avolition), functional deficits (e.g. social skills), comorbid symptoms (e.g. anxiety), or cognitive symptoms (e.g. deficits in attention, working memory, and problem-solving), which are strong predictors of functional recovery in schizophrenia [27, 28]. AR/VR holds promise as a new therapy augmenting antipsychotic medication for both positive and negative symptoms. For example, VR can be used to create controlled environments in which patients are guided through social situations to improve social functioning and life skills [29], or to design avatars, voices, and settings that approximate their perceptual disturbances to practice management strategies, guided by a therapist [29–31]. Furthermore, given that psychosis onset disproportionately affects young adults in their early 20’s, a technological, entertaining, and gaming-style intervention may be both relatable to and well-received by this population [32, 33].

Emerging literature has begun to establish that AR/VR strategies might be acceptable to patients with psychosis, but it is unclear how effective these strategies are and for what applications in patients with psychotic disorders. Therefore, the objective of this systematic review is to investigate the effectiveness of AR/VR technologies in the diagnosis and treatment of primary psychotic disorders. Through the examination of existing clinical applications of AR/VR for psychosis treatment and diagnosis, we aim to inform future research and intervention development.

## Methods

### Search Strategy

**Fig. 1:**
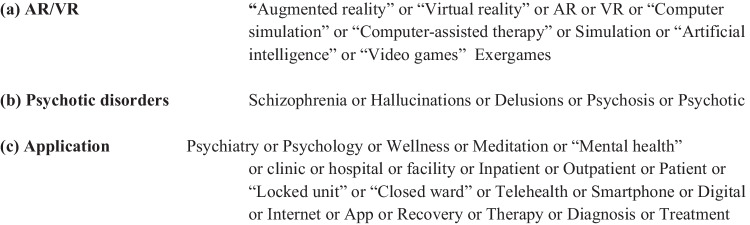
Search terms

We searched the following electronic databases for relevant studies: PubMed, PsychInfo, Embase, and CINAHL on 2/4/2020. We included three umbrella concepts in this search with related terms under each concept: 1) AR/VR (including terms such as augmented reality, virtual reality, computer simulation, computer-assisted therapy, artificial intelligence, and video games); 2) Psychotic disorders and/or symptoms (including terms such as schizophrenia, hallucinations, delusions, and psychosis); and 3) Application (including terms such as psychiatry, meditation, inpatient/outpatient, telehealth, diagnosis, and treatment). Figure 1 illustrates the search terms used. We also identified papers by reference-checking. No setting, date, or geographical restrictions were applied.

### Study Selection

We included published, peer-reviewed, original research studies written in English, including studies with human subjects of any age and a diagnosis of any primary psychotic disorder, including schizophrenia, schizoaffective disorder, schizophreniform disorder, first episode psychosis, delusional disorder, and early onset psychosis. We included populations with a psychotic disorder and other psychiatric comorbidities. We excluded book chapters, conference abstracts, study protocols, and articles not reporting on original data, such as review papers and editorials.

**Fig. 2:**
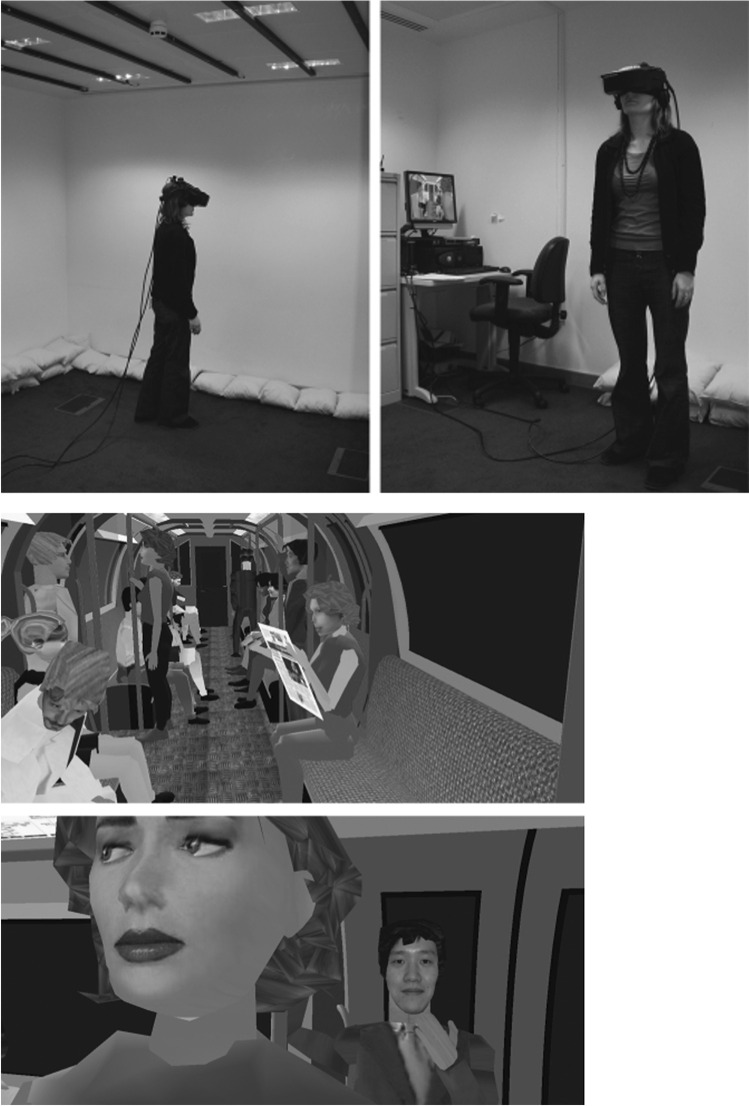
A 360° VR headset and images from within the headset [34]

We included studies using fully and partially immersive AR or VR approaches to diagnose and/or treat patients with psychotic disorders. In fully immersive VR, the user feels physically surrounded by the digital setting (e.g. 360° headset, 3D projection system onto four walls), disconnecting them from the real world and providing an engulfed sense of “being” in the task environment [7, 34]. An example of a 360° VR headset and digital setting is illustrated in Fig. 2. Partially immersive VR interventions provide a partial sense of presence by attempting to partially immerse the viewer (e.g., 180° screen or digital screen with multidimensional depth) or including two senses in an interactive experience (e.g., joystick or full body movement plus visual stimulation). We excluded non-immersive AR/VR approaches, such as computerized cognitive remediation therapy [35, 36], and those without interactive components, such as one-dimensional video/computer games.

### Data Collection

After removing duplicates, titles and abstracts were screened by two independent reviewers for compliance with inclusion and exclusion criteria. Two reviewers then independently evaluated the included full text articles for eligibility, and systematically extracted and inputted the following data into an electronic data collection spreadsheet: title, author(s), year of publication, study objectives, study design/methods, population characteristics (including primary and secondary diagnoses), baseline symptoms, type of AR/VR, results, limitations, and key insights. We defined the type of AR/VR utilized by the intervention or technology received by the study population. In both the initial abstract and full text reviews, any discrepancies between reviewers were discussed until consensus was reached, involving the first author as a third reviewer as needed.

### Data Analysis

Relationships within and between studies were explored by reviewing tabulated data and themes. Furthermore, two authors (LL and JL) independently assessed article quality and resolved disagreements via consensus, using the Effective Public Health Practice Project (EPHPP) Quality Assessment Tool for Quantitative Studies [37]. This tool with good construct validity rates the quality of quantitative studies, which are assigned a global rating of strong, moderate, or weak based on study selection bias, design, confounders, blinding, data collection methods, withdrawals, and drop-outs.

## Results

**Fig. 3:**
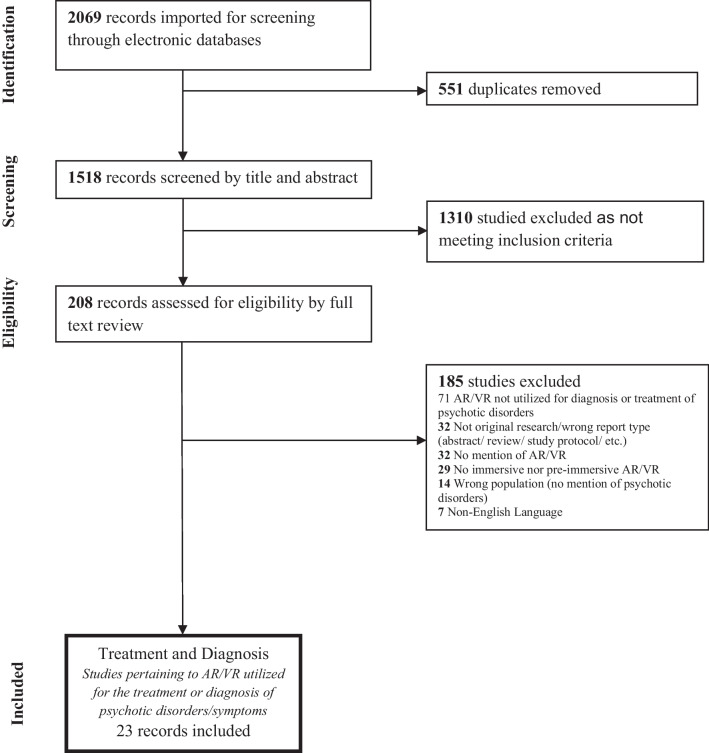
PRISMA diagram of studies reviewed

The PRISMA flow diagram of screened articles, with reasons for exclusion, is depicted in Fig. 3 [38, 39]. Our initial search resulted in 2069 articles; following the removal of duplicates, abstract screening, and full text review for eligibility, 94 original studies remained that used immersive and partially immersive VR for psychosis. No studies included participants receiving an AR-based intervention. At this stage, 71 articles were excluded because they did not examine the use of VR for diagnosis and treatment (Fig. 3). Twenty-three articles remained that studied the application of VR for diagnosis of schizophrenia (Table 1) or treatment of psychosis, targeting positive symptoms and comorbid anxiety (Table S1), resulting functional deficits including social and vocational skills (Table 2), impaired cognition (Table 3), and increasing physical fitness (Table 4). Three studies met criteria for two treatment categories [40, 41]. Only one of the 23 papers explored VR as a diagnostic tool for psychosis (Table 1) [42]. VR treatment interventions were tested in 10 randomized controlled, one non-randomized controlled, and 8 single-arm intervention trials, as well as one case control study and three small case series. These 23 studies were assessed for quality via the EPHPP Quality Assessment Tool, and are the focus of this review. The studies hailed from 14 countries: China, Korea, Japan, Israel, Germany, France, Italy, Spain, Portugal, Netherlands, UK, Canada, USA, and Brazil.

### Populations Studied

Within the 23 studies, a total of 627 individuals were enrolled, including 366 participants receiving VR interventions and 275 receiving a control (some crossing over to VR group later). Participants with the following DSM-IV/V diagnoses were studied, listed from most common to least were: schizophrenia (534); anxiety disorder comorbid with a primary psychotic disorder (45); depression symptoms comorbid with a primary psychotic disorder (43); schizoaffective disorder (22); psychotic disorder not otherwise specified (NOS) (19); delusional disorder (3). Twenty-seven participants had chronic schizophrenia [43]. The severity and stability of illness varied since participants were recruited from both inpatient (168) and outpatient (248) settings. Most studies included participants aged 18–65 (Tables 1–4); Chan et al. studied geriatric (> 60 years old) patients [43]. Study participants included men (415) and women (200). Race/ethnicity was reported in 12 studies: Asian (200); Caucasian (138); Black/African (15); other (12). Of the 5 papers that reported employment status: only 5 patients were employed; one patient had retired; and 113 patients were unemployed. Ten papers reported psychotropic medication status: unspecified antipsychotics (277); first-generation antipsychotics (39); second-generation antipsychotics (131); clozapine (10); antidepressants (36); benzodiazepines (16); and other (16).

### VR for Diagnosis of Schizophrenia (Table 1)

In a case–control study by Sorkin et al. [42], 39 individuals with schizophrenia and 21 matched healthy controls navigated a 3D virtual maze with different virtual challenges and rewards, inspired by the Wisconsin Card Sorting Test [42]. Compared to their healthy peers, participants with schizophrenia had greater difficulty with working memory, slower response times, difficulties ignoring irrelevant information, and greater consecutive and frequent errors. However, the participants with schizophrenia improved significantly more than the comparison group during the pre-experimental training session. A classification scheme, based on subjects’ VR performance profiles, was able to correctly predict a diagnosis in 85% of the patients with schizophrenia. The authors suggest that VR appears promising as a cognitive test platform to diagnose schizophrenia, in contrast to current clinical diagnostic methods [42].

### VR for Positive Symptoms (Table 1)

Four studies assessed the use of VR-assisted treatments for positive symptoms, including 3 studies examining paranoia and delusions [44–46] and one studying auditory hallucinations (AH) [47]. All 4 studies, including one single-arm and 3 randomized controlled trials (RCTs), found improved symptoms with VR treatment.

Moritz et al. [44] conducted a single-arm non-controlled trial including 33 participants with schizophrenia to test if feedback for false judgments in a VR setting attenuates delusions (a form of VR-assisted bias modification therapy) [48]. Participants maneuvered a virtual street twice (noise vs quiet scenarios), passing by avatars with varied facial expressions, after which their recall of the avatars was graded. After, patients received feedback as to whether their decision was correct. Receiving feedback on erroneous thoughts attenuated the strength of paranoid biases [44].

Two RCTs examined the use of VR exposure plus cognitive behavioral therapy (VR-CBT) to treat paranoid and persecutory delusions. Freeman et al. [45] tested a VR intervention with repeated, graded exposure with vs. without CBT for treatment of persecutory delusions [45]. Thirty individuals with non-affective psychotic disorders and significant persecutory delusions were randomized to VR exposure only vs. VR-CBT that encouraged patients to let go of safety-seeking behavior. At the beginning and end of the day, participants were asked to rate: 1) conviction in their delusion on a scale of 0% (not convinced at all) to 100% (absolutely convinced). Before starting a VR or real-world exposure, patients were asked to rate distress levels on a scale of 0 (not distressed al all) to 10 (extremely distressed). Compared to those who received VR only, individuals who received VR-CBT: travelled significantly further distances within the virtual social environment; socialized more with avatars; and exhibited large reductions in delusional conviction (22%) and real-world distress (19.6%). Pot-Kolder et al. [46] studied VR-CBT vs. treatment as usual (TAU- antipsychotic medications, and regular visits by a psychiatric nurse and doctor) for treating paranoid ideation in subjects with psychotic disorders in a single-blind RCT. VR-CBT significantly reduced paranoia and anxiety during social interactions, with sustained benefit at 6 months [46].

Only one study tested use of VR for treatment of AH using an RCT design. Du Sert et al. [47] randomized 19 subjects with schizophrenia or schizoaffective disorder experiencing persecutory AH to a VR-assisted therapy (VRT) vs. TAU (antipsychotic medications and usual meetings with clinicians). Over seven VRT sessions, participants interacted with an avatar resembling the voice and face of their most distressing persecutor. The therapist “played” the voice of the AH, using a voice-transforming technology and phrases supplied by the patient, allowing the patient to interact with their AH. VRT significantly reduced distress associated with AH as measured with Psychotic Symptoms Rating Scale (PSYRATS), decreased beliefs in the malevolence and omnipotence of the voices, and diminished general symptoms as measured with PANSS and decreased depressive symptoms, compared to TAU [47].

### VR for Anxiety (Table 1)

Three studies (one RCT, one case series, one case report) tested the application of VR to treat comorbid anxiety disorders in patients with psychotic disorders [40, 49, 50]. Fusco et al. [49] tested whether VR, with its immersive and “sense of presence” capabilities, could improve the effectiveness of progressive muscle relaxation (PMR), which decreases anxiety and improves coping in patients with schizophrenia [49, 51]. In a RCT of 22 patients with a psychotic disorder, PMR delivered via VR significantly reduced anxiety compared to standard PMR [49]. Gega et al. [50] conducted a case series, where one 60-min VR intervention was included during a 12-session course of cognitive behavioral therapy (CBT) for social phobia with 6 men with psychosis. Integration of VR allowed the therapist to manipulate exposure to feared social stimuli in real time [50]. Participants watched a 3D life-size projection of themselves interacting within a previously filmed and scripted video clip of a social scenario. Therapists observed the behavioral responses of participants confronted with a simulation of a potentially anxiety-provoking social situation and used these observations to encourage participants to engage in “real-world” exposures. Patients experienced significant improvement in social anxiety and paranoia at 24-weeks post baseline. Four of the six participants reported that the virtual environment felt “not real,” handicapping the potential usefulness of VR simulation. In a case study by Rus-Calafell et al. [40], one adult completed biweekly VR social skills training (VR-SST) sessions for 3 months and experienced improvements in social anxiety, as well as facial emotion recognition, conversation time, assertiveness, interpersonal communication, and negative symptoms [40].

### VR for Social Skills (Table 2)

**Fig. 4:**
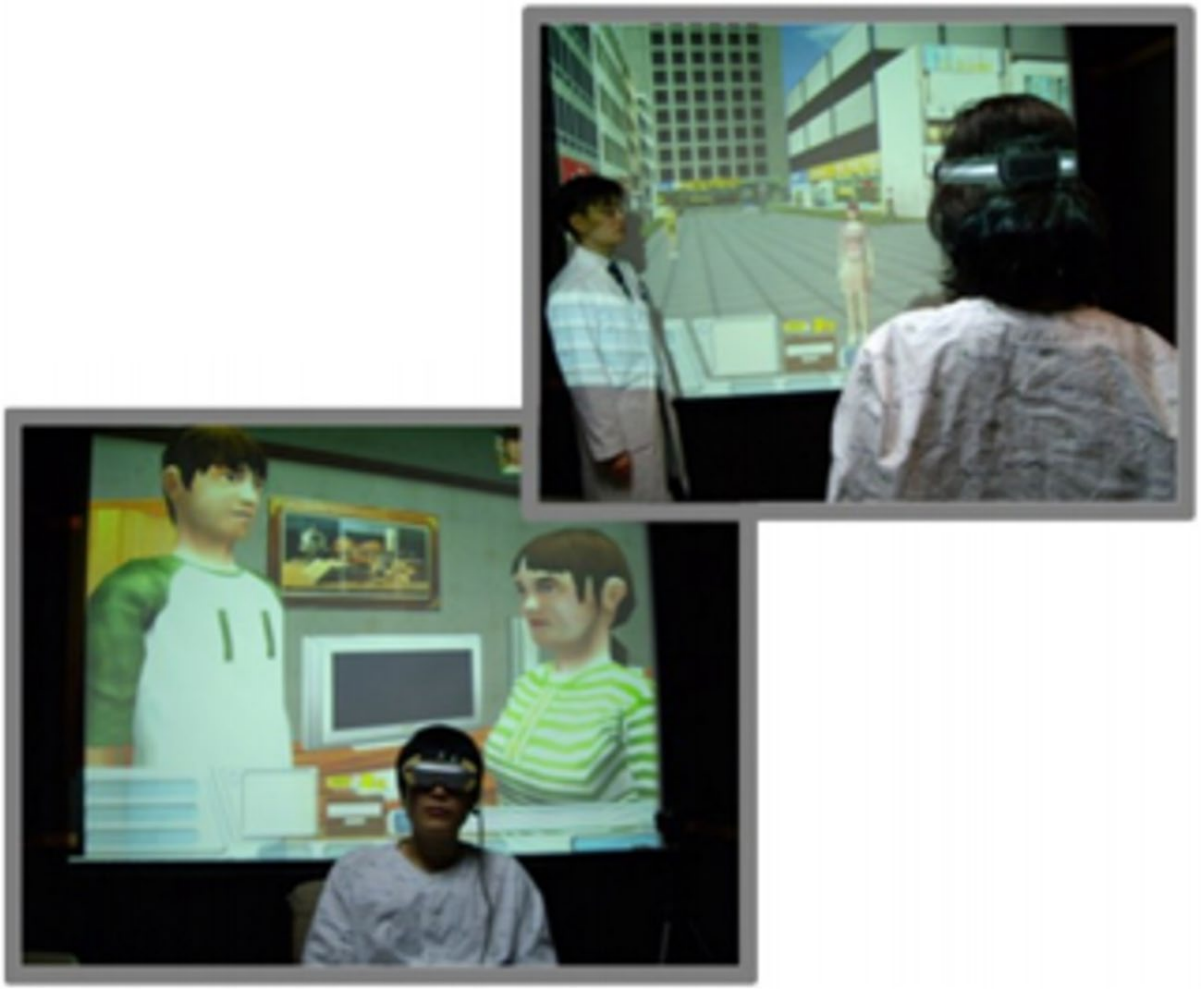
Example of social skills training within an immersive virtual reality environment [41]

Three studies (one RCT, one single-arm pilot, and one single case study) utilized VR to provide social skills training (SST) to patients with schizophrenia using integrated VR with SST-facilitated immersive exposure to social stimuli, role-plays with virtual avatars, and live therapist feedback on client behaviors to target [40, 41, 52], as depicted in Fig. 4. The most well-powered study of SST-VR for schizophrenia was a RCT of 91 inpatients who received either SST-VR or traditional SST [41]. Social skills improved in both groups. Compared to the control group, the SST-VR group displayed greater motivation and improvement in conversational skills, but SST-VR was less effective in enhancing vocal and non-verbal skills. The authors concluded that the interactive nature of VR is better suited for training conversational skills than more internal nonverbal processes. Ku et al. [52] conducted a single-arm pilot study of 10 participants with schizophrenia who completed a VR-based conversational skills program (using joysticks to initiate, navigate, and end conversations with virtual avatars); no significant correlation was found between patients’ perception of a virtual avatar and total PANSS score [52]. There were significant inverse correlations between emotional withdrawal (a subitem of the PANSS negative symptom scale) and measures of social presence and others’ perceived presence, suggesting that the more emotionally withdrawn a patient is, the less he or she felt the presence of an avatar, and the less interactive they were with the avatar. Social presence scores were negatively correlated with “silence-breaking” time, suggesting that participants who felt more present in the VR scenario were quicker to start conversations with avatars. The authors concluded that VR can be used for conversation training, but that VR use is limited by how VR is perceived. As previously mentioned in the *VR for Anxiety* section, a case study of one adult patient found improvements in social skills and social anxiety after biweekly VR-SST sessions for 3 months [40].

### VR for Vocational Rehabilitation (Table 2)

Sohn et al. [53] conducted the only study testing VR for vocational rehabilitation [53]. In a single-arm pilot study, 9 outpatients with schizophrenia received 8 sessions of one-on-one vocational rehab, in which participants were “employed” in the VR setting as supermarket or convenience store clerks, completed job-related tasks, and received live feedback. Over the course of treatment, participants exhibited significant improvements in neurocognition, general psychotic symptoms, and general social functioning.

### VR for Cognition (Table 3)

**Fig. 5:**
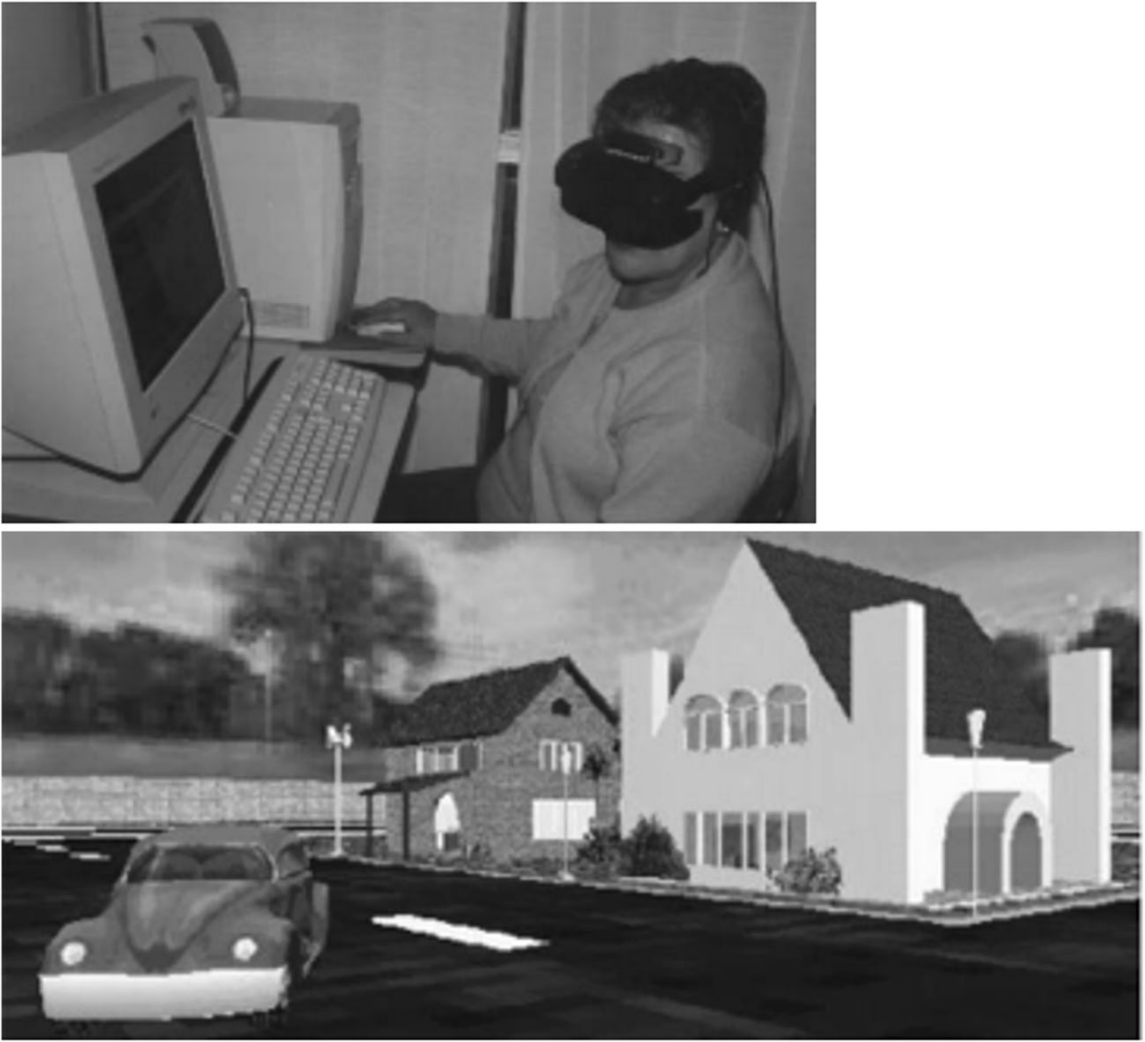
Example of cognitive training in a virtual city environment [54]

Eight studies used VR to target cognitive impairment associated with a primary psychotic disorder [43, 54–60]. Four studies (two single arm [54, 56] and two RCTs [43, 55]) used “real-world” virtual environments: virtual city [54], supermarket [54], pastoral setting [43], boutique [55], and town [56]) to train participants on everyday cognitive tasks; two RCTs tested the NeuroVR cognitive training software [57, 58]; and two studies (one RCT [59] and one controlled, single-blind crossover study [60]) tested interactive videogame aerobic exercise platforms to improve cognitive functioning. Da Costa et al. [54] pilot tested a virtual environment (VE) in which 4 patients with schizophrenia were presented with cognitive tasks in an interactive simulation in a virtual city, as depicted in Fig. 5. Of note, the authors focused on studying the usability and acceptance of VR, and not specific cognitive outcomes in this study. Participants used a head-mounted display and a computer mouse to navigate the city, enter buildings, and complete day-to-day tasks requiring a range of cognitive abilities. Participants found the virtual city enjoyable and engaging, demonstrated a strong capability to navigate and control the technological devices, and reported no adverse side effects [54]. The authors hoped to apply this environment to future studies to improve cognitive domains in patients with schizophrenia. Chan et al. [43] conducted a RCT in which 27 participants with schizophrenia and moderate cognitive impairment wore sensors and engaged in real-time interaction with objects in a virtual pastoral setting (e.g., catching beach balls) displayed on a screen in front of them. Results showed significant improvements in overall cognitive functioning and volition in participants who received the VR treatment compared to those randomized to the control group (regular occupational therapy). Tsang et al. [55] conducted a RCT of a VR based cognitive training program using a virtual boutique scenario among 75 individuals with schizophrenia. Participants in the VR training group performed significantly better than participants in the therapist-administered training group and the “Conventional Group” (no cognitive therapy) in overall cognitive functioning, and in two cognitive subscales of repetition and memory. VR participants experienced VR as more feasible, engaging, and motivating compared to conventional cognitive training in both control groups. Amado et al. [56] conducted a pilot study of a group cognitive training intervention using a virtual town setting. Eight participants with schizophrenia navigated through the 3D virtual town (one participant controlled the joystick, the rest provided instruction and feedback). Participants were given a 2D map version of the virtual town and were tasked with memorizing an itinerary, following instructions, and planning and completing various actions. After 12 weeks of training, participants improved significantly in psychosocial functioning with increased autonomy, more concrete job search plans, improved housework management. Significant improvement in the cognitive domains of attention, processing speed, working memory, and retrospective memory, was also observed [56].

Two RCTs tested the NeuroVR system, which includes a hierarchical sequence of progressively more complex tasks (e.g. picking up different types of glass bottles while following instructions via a loudspeaker) training attention and executive functioning in four distinct virtual environments (park, valley, beach, and supermarket) [57, 58]. La Paglia et al. [57] conducted a RCT with 12 outpatients with schizophrenia who completed 10 individual VR based cognitive training sessions using NeuroVR; 6 control patients completed non-VR group therapy with cognitive and social skills training. Participants in the VR group showed significant improvements on measures of general cognitive ability and executive functioning [57]. La Paglia et al. [58] conducted a second RCT of NeuroVR version 2.0 of attention training, via head-mounted displays among 15 individuals with schizophrenia. Participants in both VR and the group therapy control intervention showcased heightened performance on a measure of divided attention following training. Those in the VR group exhibited greater improvements in measures of general cognitive functioning, problem-solving, and sustained attention [58].

Kimhy et al. [59] conducted a RCT of a pre-immersive VR active video game (Xbox 360 Kinect, Your Shape Fitness evolved 2012 Ubisoft) to examine the impact of aerobic exercise on neurocognitive function and brain-derived neurotrophic factor (BDNF) regulation in 33 patients with schizophrenia. Compared to the control group (regular meetings with a psychiatrist and as-needed meetings with psychologists, RNs, social workers), the aerobic exercise group had significant improvements in physical fitness, as well as increased serum BDNF levels; the authors determined these increased levels were clinically significant and explained variance in neurocognitive functioning. The aerobic exercise group also improved significantly on measures of neurocognition compared to the control group [59]. Shimizu et al. [60] used Nintendo Wi-Fit to study VR’s effect on frontal lobe functioning in 8 Japanese outpatients with schizophrenia receiving community psychiatric services. Compared to pre-intervention, there was a significant increase in cerebral blood flow after the intervention, measured by functional near-infrared spectroscopy (fNIRS), in the dorsolateral prefrontal cortex (DLPFC), a region responsible for executive function [60]. However, there was no significant improvement on the Frontal Assessment Battery after the intervention. Participants also experienced significant improvements in bodily pain, social functioning, and role/emotional score, but not in physical functioning.

### VR for Physical Fitness (Table 4)

Six studies (one single arm study; one non-randomized controlled trial; two RCTs; one controlled, cross-over study; and one 2-case series) examined the use of VR to improve physical fitness in patients with psychotic disorders [61–64]. As mentioned above in the *VR for Cognition* section, Kimhy et al. [59] and Shimizu et al. [60] used VR-assisted physical fitness in an attempt to improve cognition, with improvements in physical fitness [17] and bodily pain [60].

Leutwyler et al. [63] examined the impact of Xbox 360 Kinect on physical activity in 20 older adults (> 55 yo) with schizophrenia or schizoaffective disorder [63]. Physical activity was measured by self-report and via Sensewear Pro Armband. Compared to pre-intervention, there was a 67% increase in the number of steps taken and a 61% reduction in sedentary activity after the intervention [63]. Leutwyler et al. [64] later conducted an RCT with 28 participants who either played an active (*n* = 13) or a sedentary (*n* = 15) video game. Walking speed improved by 0.08 m/s in the active group and by 0.03 m/s in the sedentary group. Although the change in walking speed was not statistically significant, this was reported as a clinically meaningful improvement.

Campos et al. [62] conducted a non-randomized, controlled study to evaluate feasibility and acceptability of Microsoft Xbox Kinect in 32 patients with schizophrenia. Patients were assigned to either TAU or an exercise video game intervention with animated grape harvests, runs, and stomps (culturally relevant to Porto, Portugal, which is famous for its port wine) [62]. Aerobic endurance improved in 76.9% of individuals receiving the video game intervention, and 53.8% 53.8% of patients improved in balance, flexibility, and motor coordination. The majority of the intervention group patients rated the game as satisfactory, interactive, and an acceptable form of exercise. However, most patients expressed that they would not use the game without technical assistance.

Finally, Jo et al. [61] studied the effects of Nintendo Wii-Fit on two Korean men with schizophrenia (53 and 61 years old) in a psychiatric hospital [61]. Both patients exhibited improvements in cardiovascular endurance, but not in mobility.

### Adverse Side Effects

Side effects to VR interventions appear to be minimal. There was very little mention of adverse side effects within the 23 included papers. Du Sert et al. [47] found that the first two weeks of VR treatment were the most anxiogenic. Eight studies did not mention side effects and 14 studies reported a lack of side effects from VR. Six participants within the Gega et al. [50] study perceived that the virtual environments did not feel real and did not accurately simulate the anxiety they were trying to overcome, thus believing that simulation was less useful than real-life scenarios [50].

## Discussion

In this systematic review of virtual and augmented reality for the diagnosis and treatment of psychosis, we found 23 studies testing VR interventions targeting a range of symptoms, comorbidities, and functional impairments for individuals with psychotic disorders. Although most studies were small pilot studies, more than half (15/23) included a control group and many of the interventions tested showed initial promise, suggesting that further study is warranted. Only one study examined a VR intervention as a diagnostic tool, and no studies tested AR interventions, illustrating an even bigger evidence gap in the study of AR and VR. To our knowledge, this is the first systematic review to examine AR/VR applied specifically to the diagnosis and treatment of psychotic disorders, without limiting geographical setting or publication year. We found this strategy to be critical, since identified studies were published by 20 disparate research groups hailing from 14 countries (China, Korea, Japan, Israel, Germany, France, Italy, Spain, Portugal, Netherlands, UK, Canada, USA, and Brazil).

The majority of previous publications examining the use of VR have been predominantly limited to specific phobias [21], anxiety disorders [22], PTSD [23], ASD [24], eating disorders [25], ADHD [26] and substance-use disorders [3]. Contrary to common misconceptions that engaging patients with psychosis in virtual settings with avatars or voices that resemble their hallucinations may be harmful to therapeutic progress, this review illustrates that VR methods may reduce symptoms and improve physical health [63], quality of life [60], and psychosocial functioning [41, 53], serving as an enhancement of evidence-based therapeutic and medication treatments. 

Since these studies were initially identified in 2020 for the present systematic review, the VR mental health field has continued to advance rapidly, crossing over from research to commercial offerings. This advancement has been made possible by: FDA clearance for psychiatric digital therapeutics starting Fall 2020; the rapid shift from mainly in-person mental health care to virtual visits in setting of the Covid-19 pandemic and its resulting spike in prevalence and incidence of mental health disorders; and hardware and software innovations making VR more accessible to consumers. The research publications above mention use of complex hardware with multiple components (cables, computers, headsets, projectors) that were not easily accessible to consumers; currently, the clinical VR setting utilizes wireless, portable headsets (ex- Oculus Quest) available to providers and patients via popular commercial retailers for several hundred dollars each, or as affordable as $10 via Google Cardboard utilizing an inserted smartphone. Commercial platforms now offer patients a way to treat anxiety-based disorders via such at-home, portable VR headsets. Oxford VR, based on Professor Daniel Freeman’s research, offers treatment for psychosis via its gameChange platform. We hope that continued innovations within metaverse research and resulting interventions will improve treatment options for psychosis and other serious mental illnesses.

## Limitations

The use of VR to treat psychosis is a relatively new approach and thus there are key gaps in the literature. First, the studies identified here have methodological limitations. Small, purposive sample sizes may lack cultural, social, and geographical representation and therefore limit the generalization of results to other populations [43, 46, 53, 55–58]. There is also a limitation in extrapolating results to the general public without testing scalable VR delivery systems. Additionally, at least six studies did not control for confounding variables, including presence of psychotropic medications [53, 55, 56], positive and negative symptoms [43, 55], and previous treatment [55]. Pot-Kolder et al. [46] noted technological limitations, specifically limited conversational opportunities between the user and virtual avatar. Many studies did not use an active control condition, blinded assessors, or randomized trial designs [44–47, 53]. The majority of studies also lacked follow-up data, thereby limiting the assessment of the long term effects of VR interventions [43, 44, 46, 55]. There is a variance between studies in total exposure time to VR in terms of duration of each VR session and the number of VR sessions administered; thus, the comparison between studies is not equivalent in terms of VR “dose.” Da Costa et al. [54] also reported bureaucratic and financial barriers to delivering the VR intervention.

Although there was little mention of adverse side effects in the included studies, VR has been noted to cause motion sickness including eye fatigue, headaches, nausea, and sweating in other studies [65]. Adverse side effects such as preoccupation or addiction to the virtual environment may occur with more long-term engagement in VR interventions [66]. Such side effects should be examined with larger sample sizes, longer intervention periods, and long-term follow up. While the widespread and consistent use of valid and standardized symptom scales (e.g., PANSS and BPRS) was a strength of the studies under review, the diversity of outcome measures used across studies of VR interventions within each target symptom domain made it difficult to draw conclusions across studies. Additionally, the majority of studies did not include a measure of presence. In VR literature, presence is defined broadly as a participant’s feeling of being immersed in a virtual world. Future trials in this research area may benefit from including a measure of presence to assess whether the level of presence moderates VR treatment outcomes for individuals with psychosis [67].

Of note there were no studies using AR for the treatment or diagnosis of psychotic disorders. Future research should investigate the feasibility of AR-based interventions for the treatment of psychosis. Future studies should also explore VR as a potential diagnostic tool for identifying patients with psychotic disorders. Further examination of potential adverse side effects of VR-based diagnostic tools and treatments are also needed. Future studies could also focus on the use of VR in psychosis psychoeducation and anti-stigma interventions [68, 69], and VR interventions for the diagnosis and treatment of psychotic disorders in underrepresented and minority populations for equitable access.

Finally, the current legal regulations for metaverse hardware and software use for healthcare purposes are minimal at the time of this review’s publication. It is very likely that as AR/VR becomes more regularly utilized in the research and clinical setting, legalities may involve tighter HIPPA privacy and security. Future studies could comment on how these new novel technologies evolve alongside changing regulations.

## Conclusions

The benefits of incorporating VR into existing treatments for psychosis are manifold. Virtual reality can augment existing treatments, leading to improvements in social skills, cognitive function, and physical fitness. In addition, the use of VR interventions may improve engagement in care for those who find VR interventions interesting or fun. Further, VR interventions could improve accessibility of treatment due to the convenience of use in the home setting, and relative affordability once deployed [54]. Accessibility is particularly important given that psychotic disorders disproportionately affect populations with lower socioeconomic status, and in the United States, racial and/or ethnic minority populations. However, using VR may require advanced technical and cognitive skills and greater initial hardware and software costs; these requirements may limit equitable access of the VR intervention [62]. Ideally, incorporation of VR interventions will improve clinical outcomes for patients with psychosis. More research is needed to examine effective scalability models of AR/VR, as well as decentralized clinical trials focusing on improving patient and provider experiences.

## Supplementary Information

Below is the link to the electronic supplementary material.

Supplementary file1 (XLSX 49 kb)
